# *Campylobacter* spp. in Eggs and Laying Hens in the North-East of Tunisia: High Prevalence and Multidrug-Resistance Phenotypes

**DOI:** 10.3390/vetsci9030108

**Published:** 2022-03-01

**Authors:** Manel Gharbi, Awatef Béjaoui, Cherif Ben Hamda, Narjes Alaya, Safa Hamrouni, Ghaith Bessoussa, Abdeljelil Ghram, Abderrazak Maaroufi

**Affiliations:** 1 Laboratory of Epidemiology and Veterinary Microbiology, Group of Bacteriology and Biotechnology Development, Institut Pasteur de Tunis, University of Tunis El Manar (UTM), Tunis 1002, Tunisia; manelgharbi2@gmail.com (M.G.); narjess.alaya@yahoo.fr (N.A.); safa.hamrouni@pasteur.tn (S.H.); ghaithbessoussa@hotmail.fr (G.B.); abdeljelil.ghram@pasteur.tn (A.G.); abderrazak.maaroufi@pasteur.tn (A.M.); 2 Laboratory of Bioinformatics, Biomathematics and Biostatistics, Institut Pasteur de Tunis, University of Tunis El Manar (UTM), Tunis 1002, Tunisia; cherifbenhamda@gmail.com

**Keywords:** *Campylobacter*, layer hens, eggshell, occurrence, multidrug resistance

## Abstract

Despite the importance of eggs in the human diet, and unlike other products, for which food safety risks are widely investigated, information on the occurrence of *Campylobacter* and antimicrobial resistance in eggs and layer hen flocks is lacking in Tunisia. This study was conducted to determine the occurrence of *Campylobacter* and the antimicrobial resistance in layer hens and on eggshells. Thus, 366 cloacal swabs and 86 eggshell smear samples were collected from five layer hen farms in the North-East of Tunisia. The occurrence of *Campylobacter* infection, and the antimicrobial resistance rates and patterns, were analyzed. The occurrence rates of *Campylobacter* infection in laying hens and eggshells were 42.3% and 25.6%, respectively, with a predominance of *C. jejuni* (68.4%, 81.9%), followed by *C. coli* (31.6%, 18.2%). The antimicrobial susceptibility testing revealed high resistance rates against macrolides, tetracycline, quinolones, β-lactams, and chloramphenicol, with percentages ranging from 35.5% to 100%. All isolates were multidrug resistant (MDR) and five resistance patterns were observed. These results emphasized the risk to consumer health and the need to establish a surveillance strategy to control and prevent the emergence and the spread of resistant strains of *Campylobacter* in poultry and humans.

## 1. Introduction

*Campylobacter* is one of the leading bacterial causes of food-borne diseases, presenting important challenges to public health around the world [1,2]. *Campylobacter jejuni* and *C. coli* are the major species of clinical significance, responsible for more than 95% of campylobacteriosis in humans worldwide [3]. *Campylobacter* is considered to be a commensal bacteria colonizing gut birds; however, it was shown that *C. jejuni* is responsible for damage in the intestinal mucosa of birds, leading to systemic infections with diarrhea [4,5], and could induce leg burns or podo-dermatitis during viral co-infection [6]. In addition, *C. hepaticus* is the cause of spotted liver disease in chickens [7]. 

Human campylobacteriosis, a typical food-borne illness, causes clinical cases ranging from mild symptoms to fatal outcomes, such as reactive arthritis or Reiter and Guillain–Barré Syndromes [8]. The global burden of morbidity and mortality due to *Campylobacter* spp. showed that 550 million people worldwide suffer from campylobacteriosis, with a mortality rate of about 33 million per year [2]. Campylobacters are the most frequently isolated enteric bacterial pathogens in both developed countries and low- and middle-income countries (LMICs) [9]. The genus of *Campylobacter* includes 17 species and 6 subspecies; the thermotolerant species *C. jejuni* and *C. coli* are the best known human pathogens causing human gastroenteritis [10,11].

Several studies have reported that contaminated poultry is recognized as the major source of food-related transmission of *Campylobacter* to humans, due to improper handling or consumption of raw or undercooked meat, with 50% to 80% of human campylobacteriosis cases related to chicken sources [12]. 

The chicken intestines are considered to be the main reservoir of thermophilic *Campylobacter* species [13]. These bacteria can persist in feces and litter for many days, increasing the risk of egg surface contamination [14,15], which is a potential source of many food-borne illnesses [16,17]. 

*Campylobacter* contamination of broiler chickens, carcasses and meat is well documented, but few data are available concerning eggshell contamination [18]. Indeed, while the literature on broiler chicken infections is extensive, studies on the epidemiology of *Campylobacter* species in layer chickens are very limited. Over the years, increased rates of *Campylobacter* strains resistant to the antimicrobial agents of choice (fluoroquinolones and macrolides) and the alternative therapies (gentamicin and tetracycline) have been reported, making *Campylobacter* resistant strains an emerging public health concern [19]. 

Resistance to antimicrobials is partly due to their misuse both in human and veterinary medicines [20]. Different quinolone-antibiotics have been extensively used in poultry, leading to the emergence of quinolone-resistant strains of *Campylobacter* originating from chickens and humans [20]. Recently, sitafloxacin (SIT) was proven to be effective among various fluoroquinolones-resistant pathogens including *Campylobacter*, which could be a promising drug. As a consequence of the increased resistance to quinolones throughout the world [21], erythromycin (ERY) is the recommended drug for treating human campylobacteriosis [22].

This study was conducted to investigate the occurrence of *Campylobacter* in layer hens and eggshells and to determine the antimicrobial resistance rates by analyzing the antimicrobial resistance patterns of recovered *Campylobacter* strains in the North of Tunisia. 

## 2. Materials and Methods

### 2.1. Sample Collection 

A total of 366 cloacal swabs and 86 eggshell smears were randomly collected, during the period between October 2017 and May 2018, from five laying hen farms. All farms use an intensive floor hen rearing system with bird numbers ranging from 2000 to 18,000 hens per house. The samples were taken from Lohmann and Novogen Whitehens, with ages ranging from 65 to 75 weeks.

The sampled eggs were in nesting boxes inside the houses, and the swabs were soaked and placed in Bolton broth in a refrigerated container.

All farms display similar breeding and biosecurity/biosafety protocols. The sampled farms are located in the governorates of Ben Arous and Nabeul in the North-East of Tunisia, and these areas are responsible for 40.8% of the national layer hen production [23].

### 2.2. Isolation of Campylobacter 

Upon arrival at the laboratory, samples were inoculated into Bolton Broth (Oxoid, Basingstoke, UK), containing the Bolton selective supplement for enrichment, and then incubated at 42 °C for 24 h in a microaerobic environment (5% O_2_, 10% CO_2_, and 85% N_2_), with GENbox generators (BioMérieux, Craponne, France). After enrichment, putative *Campylobacter*-positive samples were streaked on Karmali agar (Oxoid, Basingstoke, UK) and incubated under the same conditions as described above for 48 h [24]. From each sample, suspected colonies were examined for the typical morphology and motility of *Campylobacter*, under a light microscope and using the oxidase/catalase tests. Thereafter, presumed *Campylobacter* colonies were subjected to PCR analysis for genus confirmation and species identification. Confirmed *Campylobacter* isolates were conserved at −80 °C in Mueller–Hinton broth containing 25% glycerol (*v*/*v*). 

### 2.3. Identification of Thermotolerant Campylobacter

Total DNA was extracted from the cultured isolates as follows. Colonies were collected and suspended in 500 μL of TE buffer (10 mM Tris-HCl, 1 mM EDTA (pH 8.0)) and boiled for 10 min in a boiling water bath. The suspension was immediately cooled on ice for 5 min and centrifuged at 13,000× *g* for 5 min. The supernatant was recovered and used as a template for the PCR assay.

Confirmation of the *Campylobacter* genus of the presumed isolates was performed by PCR amplification of a specific fragment of the *16S rDNA* gene, using the primers described by Linton et al., (1996). Then, the isolates were identified as *C. jejuni* or *C. coli* by PCR assays based on amplification of the *mapA* and *ceuE* genes, respectively [25,26]. The sequences and origins of the three primer sets used for gene amplification are indicated in Table 1.

*C. jejuni* (ATCC 33291) and *C. coli* (CCUG 11283-T) strains were used as positive controls.

All PCR reactions contained 2.5 μL DNA template, 0.2 µM of each primer, 0.2 mM dNTP, 1X Dream Taq DNA polymerase buffer, and 1.0 U Dream Taq DNA polymerase, in a final reaction volume of 25 µL.

For genus identification, the PCR program was as follows: 5 min at 95 °C, 35 cycles consisting of 1 min at 95 °C, 1 min at 55 °C, 1 min at 72 °C, and a final extension step of 10 min at 72 °C. The same program was used for species identification, except the annealing temperature was at 59 °C. All DNA amplification reactions were carried out in a T100 thermal cycler (Bio-Rad, Marnes-La-Coquette, France). 

For visualization of PCR products, quantities of 10 μL were subjected to electrophoresis on agarose gel containing ethidium bromide, and bands were visualized with UV light.

### 2.4. Antimicrobial Susceptibility Testing 

The panel of the tested antibiotics was gentamicin (GEN: 10 μg), erythromycin (ERY: 15 μg), tetracycline (TET: 30 μg), chloramphenicol (CHL: 30 μg), nalidixic acid (NAL: 30 μg), ciprofloxacin (CIP: 5 μg), ampicillin (AMP:10 μg), and amoxicillin/clavulanic acid (AMC: 10/20 μg). *Campylobacter* isolates were tested against the eight selected antibiotics by the disk diffusion method, as recommended by the European Committee on Antimicrobial Susceptibility Testing [27].

The bacterial isolates were cultivated on Karmali plates for 48 h. A bacterial suspension was prepared for each isolate and adjusted to 0.5 MacFarland. A volume of 0.1 mL was spread onto a Mueller–Hinton agar plate and antibiotic discs were then applied. The diameter of the inhibitory zone was measured after cultivation for 24 h at 37 °C as previously described. Results concerning AMP, AMC, CIP, ERY, GEN, and TET were evaluated following interpretive criteria provided by the EUCAST-2017 [27]. For CHL and NAL, we used the resistance breakpoints of enteric bacteria in the family *Enterobacteriaceae* because there are no breakpoints that are specific for *Campylobacter.*

### 2.5. Data Analysis 

All the data collected within the present study were analyzed using R software, a language and an environment for statistical computing [28]. The antimicrobial resistance analyses were performed by means of a Chi-square statistic (*p* < 0.05) [29]. This test is a non-parametric tool designed to compare frequency counts between two groups of different sample sizes; the selection criteria for significantly prevalent variance was a stringent *p*-value of 0.001 or less.

## 3. Results

### 3.1. Occurrence of Campylobacter in Layer Hens and Eggshell Samples

Out of the 366 cloacal swab samples, 43% (155/366) were positive for *Campylobacter* spp. Overall, 106 isolates were assigned as *C. jejuni* (68.4%) and 49 as *C. coli* (31.6%). The occurrence of *Campylobacter* contamination on eggshells was 25.6% (22/86), with the predominance of *C. jejuni*, which showed a percentage of 81.8% (18/22), followed by *C. coli* with 18.2% (4/22). The occurrence of contamination ranged from 20% to 100% per flock and from 29% to 47.4% per governorate (*p* < 001). In the region of Nabeul, the occurrence of *Campylobacter* was 47.4% (126/266), while in the Ben Arous region, it was 29% (29/100). 

### 3.2. Antimicrobial Susceptibility 

All isolates were resistant to tetracycline, erythromycin, nalidixic acid, ciprofloxacin, and chloramphenicol. Regarding the β-lactams, a very high resistance rate (85.8%) was noted within strains against ampicillin; 98% of *C. coli* and 80% of *C. jejuni* were resistant (Table 2). 

The percentage of resistant isolates to amoxicillin/clavulanic acid was 43% in *C. coli* vs. 18% for *C. jejuni*. The lowest rate of resistance was found for gentamicin with 1.9% for *C. jejuni* isolates and 0% for *C. coli* isolates. The resistance percentages in *C. jejuni* and *C. coli* isolated from laying hens are shown in Table 2. 

Multidrug-resistance was detected among all *Campylobacter* isolates, and resistance profiles including 4 and 5 antibiotic classes were observed in 11.6% and 88.4% of strains, respectively. Five antimicrobial resistance patterns were found for all *Campylobacter* isolates (Table 3), with a predominance (43.2%) of the combination “AM-AMC-NAL-CIP-ERI-TET-CHL”.

Most of the *C. jejuni* isolates (55%) belonged to this group, as compared to the *C. coli* isolates (18%). The pattern "AM-NAL-CIP-ERI-TET-CHL” was detected in 38.7% of the isolates. The majority of *C. coli* strains were multidrug-resistant; only one *C. coli* isolate showed the specific pattern “AM-NAL-CIP-ERI-TET-CHL”.

## 4. Discussion

Compared to broiler chickens, laying hens showed a higher frequency of *Campylobacter* colonization [12,30]. When infected, the laying hens excrete large quantities of *Campylobacter* cells; therefore, their droppings represent an important source of contamination in poultry and animal farms [30]. Meat from spent laying hens is not commonly consumed; however, in Tunisia, high meat yields from such hens is marketed. Besides, there are few published data reporting the presence of *Campylobacter* in laying hen flocks. For these reasons, it was interesting to carry out this work and study the occurrence of *Campylobacter* in laying hen flocks to investigate the potential risk of *Campylobacter* infections for consumers. 

Our results revealed an occurrence of 43% (155/366) of *Campylobacter* spp. in layer hen farms, which is higher than that reported in our previous study in broilers (22.4%) [31]. Both broilers and laying hens could harbor campylobacters at high percentages in the gut at the slaughter age. However, the laying hens were raised for longer periods, which allowed the persistence and the widespread nature of infections. Nevertheless, the temporal dynamics of *Campylobacter* spp. colonization in laying hens is not yet well-understood [32]. When comparing our result (43%) with other reports, it was higher than those reported in Greece (13.3%) [33] and Australia (11%) [17], but lower than those observed in Finland (86%) [34], Italy (65%) [35], and Sri Lanka (64%) [36]. 

After identification of the isolated *Campylobacter* strains, a predominance of *C. jejuni* (67%) over *C. coli* species (33%) was noted; this is in agreement with our previously reported results in broiler flocks [31]. Such a difference has been described by several studies [33,37] and similar data were reported by the EFSA in 2016, with a predominance of *C. jejuni* (60%) over *C. coli* (40%). 

Despite the evidence that the consumption of contaminated chickens’ meat is responsible for an important percentage of human campylobacteriosis cases, the involvement of other poultry products such as eggs has not yet been studied in Tunisia. A better understanding of the role of eggs in the spread of *Campylobacter* has become necessary. Thus, one of the objectives of our study was to investigate the occurrence rate of thermotolerant *Campylobacter* on eggshells. These data could be useful for the risk assessment of human campylobacteriosis caused by the consumption of undercooked eggs, the consumption of food produced with raw eggs, or by handling eggs.

The eggshell contamination can occur in the hen’s reproductive tract or by feces after laying. Thus, subsequent contamination of egg products with *Campylobacter* spp. could be generated [38]. Our results showed that out of 86 eggshell samples, 22 (26%) were positive for *Campylobacter*. This rate is higher than that reported in Malaysia, (12%) [39], Germany (4.1%) [40], and Trinidad (1%) [41]. However, this rate remains lower than reported in Japan, which was 36% [42]. This high contamination rate could be a potential source of egg product contamination during the production process, especially for cracked eggs [40,42]. Indeed, one contaminated egg might be enough to contaminate a whole batch of unpasteurized liquid eggs, which could be a real potential risk for consumer health [33] Out of the positive samples, 82% (18/22) were identified as *C. jejuni* and 18% (4/22) as *C. coli,* showing the dominance of *C. jejuni* species, as described in Egypt [43]. 

Another important aspect that has likely contributed to the failure of controlling and therefore eradicating *Campylobacter* contamination in poultry flocks is the emergence of antimicrobial-resistant strains. Indeed, as with all infectious diseases of bacterial origin, the therapy of *Campylobacter* is essentially based on the use of antibacterial molecules. The emergence of resistant strains limits the efficiency of these drugs and causes therapeutic failures in animals and the spread of resistant strains in humans. 

In general, erythromycin and ciprofloxacin are the recommended antibiotics for the treatment of campylobacteriosis in humans [44], whereas tetracyclines and beta-lactams, which are used to treat intestinal infections, are not generally recommended in campylobacteriosis cases [45,46]. On the other hand, gentamicin was proven in vitro to have good antimicrobial activity and may be considered as an alternative treatment [47].

In our study, we tested eight antibiotics that are commonly used in poultry farms in Tunisia [31]. The results showed high rates of resistance in *C. coli* and *C. jejuni* strains to erythromycin, tetracycline, ciprofloxacin, nalidixic acid, and chloramphenicol. However, lower rates were observed for ampicillin, amoxicillin/clavulanic acid, and gentamicin. Although most antibiotics are prohibited during the laying period, the use of antibiotics during the incubation and growth periods is allowed for laying hens. During growth and development, the acquisition of antibiotic resistance genes is likely important [48]. 

The high antimicrobial resistance rates could be related to the excessive use of antibiotics in chicken farms to control bacterial infections, as well as the use of growth promoters. Indeed, several studies have shown a clear positive association between the use of fluoroquinolones in poultry production and the emergence of resistant *Campylobacter* strains in poultry and humans [49,50,51]. In countries prohibiting the use of fluoroquinolones in poultry production, such as Australia and Nordic European countries, low rates of resistant *Campylobacter* were found in chickens and humans [52]. 

On the other hand, the Horizontal Gene Transfer (HGT) plays a key role in AMR acquisition. The HGT is even more frequent in microorganisms sharing similar mobilomes, and is more likely in the gut-associated microorganisms [53].

Comparing the results of this study in laying hens with broilers [31], a difference in the resistance rates of isolated strains, as compared to nalidixic acid, was found. Indeed, it was noted that all *Campylobacter* strains isolated from laying hens were resistant, whereas only 46.2% of strains from broilers were resistant to nalidixic acid in broilers. 

Even though the use of chloramphenicol is prohibited in our country, all isolates were resistant. Interestingly, none of the sampled farms had used this drug; however, they used florfenicol as a broad-range antibiotic. On the basis of this observation, we can explain the resistance against chloramphenicol as a combined acquired resistance against florfenicol/chloramphenicol [54].

On the other hand, the low rates of gentamicin resistance found in *Campylobacter* isolates from laying hens/eggs and previously from broiler chickens (1% and 12.9%, respectively) were probably related to its infrequent use in poultry production. These resistance rates are consistent with previous studies reporting a low level of gentamicin resistance in *C. jejuni* isolated from chicken meat [51,55]. 

To a lesser extent, the rates of β-lactams resistance of *Campylobacter* isolated from layers were 85.8% for ampicillin and 35.5% for amoxicillin/clavulanic acid. These rates were higher than those reported in our previous study in broilers [31]. When looking the resistance rates within species, we noted a significant difference between ampicillin (80% versus 98%) and amoxicillin/clavulanic acid (43% versus18%), in *C. jejuni* and *C. coli*, respectively. Similar alarming resistance rates with regard to β-lactams were reported in several countries, such as Algeria (100%) [56] and Italy (100%) [57]. Even though β-lactams are not used in the treatment of campylobacteriosis in humans, the emergence of extended-spectrum beta-lactamase (ESBL)-producing *Campylobacter* strains could be a source of ESBL-gram-negative bacteria dissemination. It is noteworthy to remember that ESBL is an emerging global health threat and is associated with high mortality worldwide [58].

All *Campylobacter* isolates were identified as multidrug-resistant, with patterns including resistance to more than three antibiotics, as described in our previous results [28]. Several studies have shown that the emergence of multidrug-resistant isolates in animals represents a significant problem in Tunisia [59]. This worrying phenomenon is further complicated by the lack of an effective national antimicrobial surveillance system in husbandry.

## 5. Conclusions

Our study reported the occurrence of *Campylobacter* spp. in laying hen farms and on eggshells in Tunisia, and described AMR in the isolated *C. jejuni* and *C. coli* strains. The high antimicrobial resistance rates with multidrug-resistant strains’ emergence should be taken into consideration. Particularly, resistance to fluoroquinolones and macrolides is alarming since they are the drugs of choice. Our findings are of great concern considering that poultry are the major source of human campylobacteriosis and antimicrobial-resistant strains could be easily transmitted to humans via the food chain. Therefore, the assessment and monitoring of *Campylobacter* spp. infection in poultry flocks and AMR surveillance is needed to protect public health.

## Figures and Tables

**Table 1:** Primer sequences used for *Campylobacter* spp. identification and expected amplicon sizes.

Genes	Primer Sequences 5’–3’	Tm (°C)	Size (pb)	References
*ARNr 16S*	F: GGATGACACTTTTCGGAGC R: CATTGTAGCACGTGTGTC	52	816	Linton et al., (1996)
*mapA*	F: CTATTTTATTTTTGAGTGCTTGTG R: GCTTTATTTGCCATTTGTTTTATTA	52	589	Stucki et al., (1995)
*ceuE*	F: ATTGAAAATTGCTCCAACTATG R: GATTTTATTATTTGTAGCAGCG	52	462	Gonzalez et al., (1997)

**Table 2:** Antimicrobial resistance rates in *Campylobacter* isolates.

Antibiotic Classes	Antibiotics	Sensitivity	Resistance	*C. jejuni* (*n =* 106)	*C. coli* (*n =* 49)	Total (*n* = 155)
		(≥*S*)	(*R*<)	(%)	(%)	(%)
β-lactams	Ampicillin	19	14	80	98 *	85.8
	Amoxicillin/clavulanic acid	19	14	43 *	18	35.5
Fluoroquinolones	Ciprofloxacin	26	26	100	100	100
	Nalidixic Acid	19	14	100	100	100
Macrolides	Erythromycin	20	20	100	100	100
Tetracyclines	Tetracycline	30	30	100	100	100
Phenicols	Chloramphenicol	17	17	100	100	100
Aminoglycosides	Gentamicin	17	17	1.9	0	1

* Significant difference between the two species (*p* < 0.05).

**Table 3:** Multidrug resistance profiles of *Campylobacter jejuni* and *Campylobacter coli*.

Multidrug Resistance Profiles	*C. jejuni*		*C. coli*		Total	
	(*n* = 106) *%*		*(n* = 49) %			
	*n*	%	*n*	%	*n*	(%)
AM-AMC-NAL-CIP-ERI-TET-CHL	58	55%	9	18%	67	43.22%
AM-NAL-CIP-ERI-TET-CHL	20	19%	40	82%	60	38.7%
AM-CIP-ERI-TET-CHL	10	9%	0	0%	10	6.45%
NAL-CIP-ERI-TET-CHL	12	11%	0	0%	12	7.74%
AM-ERI-TET-CHL	6	6%	0	0%	6	3.87%

*n*: number; AM: Amoxicillin; AMC: Amoxicillin/clavulanic acid; CIP: Ciprofloxacin; NAL: Nalidixic Acid; ERY: Erythromycin; TET: Tetracycline; CHL: Chloramphenicol; GEN: Gentamicin.

## Data Availability

All the data supporting reported results are involved in this manuscript.
